# Correction: Adaptation to Spanish and psychometric study of the Flow State Scale-2 in the field of musical performers

**DOI:** 10.1371/journal.pone.0233006

**Published:** 2020-05-05

**Authors:** Laura Moral-Bofill, Andrés Lópezdelallave, Mª. Carmen Pérez-Llantada, Francisco Pablo Holgado-Tello

## Notice of republication

This article was republished on April 22, 2020 to remove a Supporting Information file that was incorrectly included in the originally published article. Please download this article again to view the correct version.
