# Supplementary material for: Adaptation to Spanish and psychometric study of the Flow State Scale-2 in the field of musical performers
Source: PLoS One. 2020 Apr 2;15(4):e0231054. doi: 10.1371/journal.pone.0231054 (PMC7117763; doi:10.1371/journal.pone.0231054)
Supplement: S1 File — (PDF) [file pone.0231054.s001.pdf]

Polychoric Correlations Matrix

	V1	V2	V3	V4	V5	V6
V1	1.000					
V2	0.275	1.000				
V3	0.476	0.619	1.000			
V4	0.352	0.308	0.454	1.000		
V5	0.108	0.006	-0.080	-0.019	1.000	
V6	0.381	0.598	0.660	0.432	0.087	1.000
V7	0.410	0.257	0.396	0.292	0.141	0.413
V8	0.432	0.609	0.601	0.399	-0.045	0.527
V9	0.482	0.610	0.720	0.483	-0.039	0.685
V10	0.380	0.290	0.496	0.822	-0.046	0.433
V11	0.120	0.089	0.055	0.026	0.772	0.163
V12	0.388	0.542	0.623	0.418	0.103	0.824
V13	0.427	0.235	0.355	0.293	0.098	0.394
V14	0.366	0.851	0.649	0.389	0.043	0.660
V15	0.468	0.602	0.839	0.504	-0.084	0.682
V16	0.370	0.293	0.474	0.748	0.012	0.437
V17	0.102	0.175	0.090	0.097	0.771	0.212
V18	0.390	0.603	0.636	0.446	0.105	0.822
V19	0.510	0.298	0.442	0.361	0.088	0.440
V20	0.342	0.848	0.634	0.371	0.061	0.660
V21	0.468	0.616	0.770	0.508	-0.060	0.712
V22	0.390	0.330	0.537	0.784	-0.052	0.503
V23	0.091	0.168	0.086	0.087	0.728	0.245
V24	0.363	0.569	0.618	0.407	0.074	0.854

	V7	V8	V9	V10	V11	V12
V7	1.000					
V8	0.406	1.000				
V9	0.466	0.667	1.000			
V10	0.328	0.470	0.545	1.000		
V11	0.246	0.064	0.057	0.028	1.000	
V12	0.437	0.533	0.679	0.441	0.188	1.000
V13	0.654	0.370	0.431	0.337	0.196	0.439
V14	0.328	0.667	0.688	0.394	0.095	0.620
V15	0.436	0.620	0.826	0.560	0.018	0.672
V16	0.327	0.432	0.509	0.806	0.053	0.406
V17	0.276	0.102	0.091	0.070	0.852	0.244
V18	0.423	0.559	0.702	0.463	0.165	0.832
V19	0.627	0.445	0.517	0.434	0.177	0.468
V20	0.311	0.682	0.663	0.392	0.129	0.635
V21	0.402	0.616	0.794	0.550	0.045	0.696
V22	0.357	0.470	0.568	0.846	0.011	0.483
V23	0.281	0.101	0.097	0.053	0.796	0.273
V24	0.382	0.542	0.680	0.425	0.147	0.864

	V13	V14	V15	V16	V17	V18
	--------	--------	--------	--------	--------	--------
V13	1.000					
V14	0.315	1.000				
V15	0.437	0.674	1.000			
V16	0.398	0.373	0.565	1.000		
V17	0.167	0.180	0.100	0.138	1.000	
V18	0.375	0.684	0.686	0.423	0.258	1.000
V19	0.789	0.382	0.519	0.472	0.209	0.461
V20	0.264	0.897	0.660	0.366	0.215	0.700
V21	0.417	0.693	0.812	0.541	0.080	0.688
V22	0.387	0.424	0.613	0.838	0.069	0.492
V23	0.243	0.178	0.110	0.129	0.882	0.273
V24	0.371	0.654	0.652	0.399	0.206	0.909

	V19	V20	V21	V22	V23	V24
	--------	--------	--------	--------	--------	--------
V19	1.000					
V20	0.344	1.000				
V21	0.516	0.706	1.000			
V22	0.462	0.431	0.606	1.000		
V23	0.283	0.204	0.099	0.089	1.000	
V24	0.446	0.666	0.687	0.488	0.250	1.000
