# Supplementary material for: Adaptation to Spanish and psychometric study of the Flow State Scale-2 in the field of musical performers
Source: PLoS One. 2020 Apr 2;15(4):e0231054. doi: 10.1371/journal.pone.0231054 (PMC7117763; doi:10.1371/journal.pone.0231054)
Supplement: Attachment — Submitted filename: respond to reviewers_2_2.docx [file pone.0231054.s003.docx]

Additional Editor Comments (if provided):

1.- Add empirical information in the abstract (eg. reliability).

We have included some global fit indexes for the model of 6 related factor and the second order factor model. Also, the alphas range of the dimensions have been included. The new abstract is:

“Flow is a positive and optimal state of mind, during which people are highly motivated and absorbed in the activity they are doing. It is an experience that can occur in any area of life. One of the measurement instruments which is most commonly used to evaluate this construct is the Flow State Scale-2 (FSS-2). This instrument has been used in different languages, mainly in the field of sport. In this research work, the FSS-2 has been translated into Spanish and administered to 486 musicians from different regions of Spain in order to examine the psychometric properties. A version which uses six dimensions from the original questionnaire has been used - those that constitute the experience of flow - and four alternative models have been analysed (Six related factors model, two second order factor models and a bifactor model).The results revealed that the dimension time could be controversial and more research could be needed. In general terms, the six-factor model (RMSEA = .04; GFI = .99; AGFI = .99) and a second-factor one (RMSEA = .033; GFI = .99; AGFI = .99) are solutions consistent with previous studies and show that the questionnaire can be considered a reliable (Alphas of the dimensions range from .81 to .94; Omegas from .86 to .97; and mean discrimination of the dimensions from .64 to 88) and useful tool, both in clinical and educational contexts, as well as an instrument for the evaluation of this construct in future research. However, the results of this study also suggest that flow can be explored in greater depth in musicians, taking into account that the writing of the original items was based on the experience of athletes and that the role of time in flow needs to be investigated”.

2.- APA style: better talk about persons, not subjects (see participants section).

Thank you very much for your advice. The use of persons has been corrected along the text.

3.- Used only two decimals (see method).

Done

4.- Add SPSS reference.

Done

5.- Please, explain all CFA models tested.

In order to facilitate the reading, we have included a brief description after each model label. For example, if we refer model 1, in brackets we have include “six related factors”. The same for the rest of model labels. The theoretical sense of each model and the references that support them are described in the text but is true that the reading was difficult only with the model labels. Also, we have tried to explain better the models.

6.- Please test one factor model.

According to the reviewer suggestion we have examined the one factor model. The fit indexes are the following:

Degrees of Freedom = 254

Normal Theory Weighted Least Squares Chi-Square = 6700.39 (P = 0.0)

Satorra-Bentler Scaled Chi-Square = 3722.06 (P = 0.0)

Chi-Square Corrected for Non-Normality = 26692.64 (P = 0.0)

Estimated Non-centrality Parameter (NCP) = 3468.06

90 Percent Confidence Interval for NCP = (3274.39 ; 3669.03)

Minimum Fit Function Value = 10.31

Population Discrepancy Function Value (F0) = 7.97

90 Percent Confidence Interval for F0 = (7.53 ; 8.43)

Root Mean Square Error of Approximation (RMSEA) = 0.18

90 Percent Confidence Interval for RMSEA = (0.17 ; 0.18)

P-Value for Test of Close Fit (RMSEA < 0.05) = 0.00

Expected Cross-Validation Index (ECVI) = 8.77

90 Percent Confidence Interval for ECVI = (8.32 ; 9.23)

ECVI for Saturated Model = 1.38

ECVI for Independence Model = 63.42

Chi-Square for Independence Model with 276 Degrees of Freedom = 27540.03

Independence AIC = 27588.03

Model AIC = 3814.06

Saturated AIC = 600.00

Independence CAIC = 27709.90

Model CAIC = 4047.63

Saturated CAIC = 2123.29

Normed Fit Index (NFI) = 0.86

Non-Normed Fit Index (NNFI) = 0.86

Parsimony Normed Fit Index (PNFI) = 0.80

Comparative Fit Index (CFI) = 0.87

Incremental Fit Index (IFI) = 0.87

Relative Fit Index (RFI) = 0.85

Critical N (CN) = 37.15

Root Mean Square Residual (RMR) = 0.19

Standardized RMR = 0.19

Goodness of Fit Index (GFI) = 0.86

Adjusted Goodness of Fit Index (AGFI) = 0.84

Parsimony Goodness of Fit Index (PGFI) = 0.73

The fit indices are not adequate, may be, because the construct respond to the structure of the theoretical position presented in the paper. Given these results we understand that present this model has not interest and, probably, will make more difficult the reading to potential readers. Nonetheless if the reviewer consider that could be of interest present this model in the paper, please, let us know it.

7.- Please check typo: bifactorial model (bifactor model).

Done

8.- Table 3. Add p values.

According to reviewer suggestion we have included an asterisk in each significant parameter, as is usual reporting results of SEM (a note in each table has been included to explain this issue). We have not included the exact p value because most of them are under .01, then we will need more than two decimals and the tables will contain a lot of information. But the relevant information about the significance of the parameters has been added.

9.- CFA: add BIC values and IC 90% RMSEA.

According to reviewer suggestion, we have included BIC in the table 2 and the IC for RMSEA along the text where the fit indices of each model are commented.

10.- Table 4, add p values.

we have included an asterisk in each significant parameter

11.- Please, add CFA measurement invariance analyses.

In all probability the reviewer is considering one of the key elements of an adequate study of validity. In this sense, invariance analysis is necessary to obtain empirical evidences about the construct validity of the scale. If we are considering different populations or groups, and if we suspect that could be found differences in how these groups understand the measured concept, an invariance analysis should be done in order to guarantee that we are measuring with the same conditions (concepts, reliability) between samples. In this research we have not considered conjectures about differences among defined groups as gender, age, instruments, or years of practice, for example. As the reviewer indicate, this could be an interesting issue to be examine, however in this paper, as a first step, our objective was to adapt the FSS-2 to Spanish and to look at the psychometric properties of the instrument in a population composed specifically of musicians. An adequate analysis of the invariance exceed the objective and extension of this paper (another different paper could be done), because an adequate invariance analysis could be extensive in results and discussion if variance is found and firstly we must review the literature to investigate in which variables the multigroup perspective should be considered (gender, instrument, years of practice,…).

In order to recognize the importance of this issue in the limitations we have added the following text: “…in this sense in order to obtain more validity evidences and be able to generalised the model tested into different groups (gender, instruments used, years of experience,…) an invariance analysis should be explore in forthcoming researches”

Finally, a technical limitation to carry out this kind of analysis is that given the complexity of the tested models the sample size do not is big enough to split it by any moderator variable as gender for example.

12.- Please, add information about the psychometric properties of: Challenge-skill balance (balance); Clear goals (goals); Unambiguous feedback (feedback).

According with this suggestion the alphas, omegas and mean discrimination of these dimensions has been added in the instrument section. The new paragraph is:

“…The remaining 3 scales of the FSS-2 that were not included in the EFIM were used as criteria, as they correspond to the dimensions that are necessary conditions in order to generate the flow state. These are: Challenge-skill balance (balance) (in this sample: Alpha = .75; Omega = .83; mean discrimination = .59); Clear goals (goals) (Alpha = .90; Omega = .90; mean discrimination = .78); Unambiguous feedback (feedback) (Alpha = .86; Omega = .87; mean discrimination = .56).”
